# Economic Friendly ZnO-Based UV Sensors Using Hydrothermal Growth: A Review

**DOI:** 10.3390/ma14154083

**Published:** 2021-07-22

**Authors:** Liguo Qin, Fagla Jules Mawignon, Mehboob Hussain, Nsilani Kouediatouka Ange, Shan Lu, Mahshid Hafezi, Guangneng Dong

**Affiliations:** Key Laboratory of Education Ministry for Modern Design and Rotor-Bearing System, Institute of Design Science and Basic Components, School of Mechanical Engineering, Xi’an Jiaotong University, Xi’an 710049, China; mawignon@stu.xjtu.edu.cn (F.J.M.); mehboob@stu.xjtu.edu.cn (M.H.); angensilani@stu.xjtu.edu.cn (N.K.A.); Shanlu@stu.xjtu.edu.cn (S.L.); mahshid.hafezi@stu.xjtu.edu.cn (M.H.); donggn@xjtu.edu.cn (G.D.)

**Keywords:** zinc oxide nanostructures, hydrothermal, localized heat growth, seed patterned growth, growth critical parameters, UV sensors

## Abstract

Ultraviolet (UV) sensors offer significant advantages in human health protection and environmental pollution monitoring. Amongst various materials for UV sensors, the zinc oxide (ZnO) nanostructure is considered as one of the most promising candidates due to its incredible electrical, optical, biomedical, energetic and preparing properties. Compared to other fabricating techniques, hydrothermal synthesis has been proven to show special advantages such as economic cost, low-temperature process and excellent and high-yield production. Here, we summarize the latest progress in research about the hydrothermal synthesis of ZnO nanostructures for UV sensing. We particularly focus on the selective hydrothermal processes and reveal the effect of key factors/parameters on ZnO architectures, such as the laser power source, temperature, growth time, precursor, seeding solution and bases. Furthermore, ZnO hydrothermal nanostructures for UV applications as well as their mechanisms are also summarized. This review will therefore enlighten future ideas of low-temperature and low-cost ZnO-based UV sensors.

## 1. Introduction

Ultraviolet (UV) light provides a special benefit on the well-being of humans by killing microorganisms. However, higher exposure has been reported to cause side effects such as skin cancer, cataracts or immune system suppression. Therefore, sensors that possess the ability of efficiently detecting UV signals have attracted huge attention [1,2,3]. These UV sensors were divided into two groups, including vacuum UV sensors and solid-state UV sensors [4,5,6]. Vacuum UV sensors are based on photomultiplier tubes and their derived devices, whereas solid-state UV sensors are based on semiconductor materials [4]. Compared to solid-state UV sensors, vacuum UV sensors have some disadvantages such as large size, high power consumption, low quantum efficiency, high pressure, low-temperature working conditions and high cost [7]. Therefore, solid-state UV sensors are the new focus for UV technology [4,8,9,10,11].

In the last few decades, semiconducting metal oxide nanoscale materials were the most likely candidates for electronic, optical, biomedical and thermal applications. They were widely used in UV lasers, sensors, field-effect transistors, field emission devices, energy harvesters, light-emitting sources, phonic devices and nanogenerators [3,4,12,13,14,15,16,17,18,19,20,21,22]. These metal oxide materials include zinc oxide (ZnO), nickel oxide (NiO), titanium oxide (TiO_2_), copper oxide (CuO), tin oxide (SnO_2_), iron oxide (Fe_2_O_3_), indium oxide (In_2_O_3_), tungsten trioxide (WO_3_) and vanadium oxide (V_2_O_3_) [1,4,14,17,19,23,24,25,26,27,28]. Among all of these materials, ZnO has gained considerable interest due to its fascinating unique properties, as mentioned in Table 1. Its properties include a direct large bandgap (3.37 eV), huge excitation binding energy (60 meV), excellent electron mobility (1 to 200 cm^2^/V. s) and huge piezoelectric coefficient (d_33_~12 pm/V) [13,16,17,23,29,30,31]. Excellent biocompatibility, biodegradability and chemical stability, as well as amazing electrical and optical properties are also some great characteristics of ZnO [12,13,15,17,20,25,32,33]. Meanwhile, various morphologies of ZnO nanomaterials have been investigated, such as nanoparticles, nanowires, nanoneedles and nanotubes, which accordingly expand their applications in various fields [12,13,14,17,19,34].

ZnO is available in three crystalline structures, including wurtzite, zinc blende and rock salt. Wurtzite structure is a two-lattice parameter-based hexagonal unit cell with a = 0.3296 nm and c = 0.52065 nm [13,18,40]. ZnO wurtzite is stable under ambient conditions, but it is transformed into rock salt at relatively high pressure (approximately 10 GPa) [18,41]. The zinc blende can only be obtained at its stable phase on cubic substrates [20,41].

**Figure 1:**
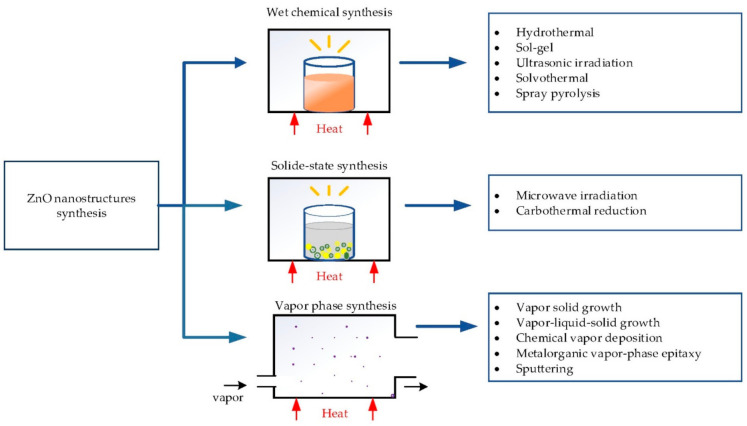
The different synthesis methods of ZnO nanostructures.

Various methods have been established to synthesize ZnO nanostructures. These methods can be divided into three groups as follows: (a) wet-chemical synthesis, (b) solid-state synthesis and (c) vapor-phase synthesis [41,42,43]. Wet chemical synthesis is carried out at low temperatures (˂200 °C) either in aqueous solution, organic solution or a mixture of both. It includes hydrothermal [23,32,33,41,43,44,45,46,47,48], sol-gel [19,49,50,51], solvothermal [52], ultrasonic irradiation growth in solution [41] and spray pyrolysis [28,53]. Solid-state synthesis includes microwave irradiation [41,42] and carbothermal reduction [13,42]. Vapor-phase synthesis is generally carried out at high temperatures (from 500 to 2000 °C). Vapor-solid growth [48], vapor-liquid-solid growth (VLS) [18,48,54], chemical vapor deposition (CVD) [17,18,20,55,56,57], metalorganic vapor-phase epitaxy (MOVPE) [17,58], atomic layer deposition (ALD) [13] and sputtering [19,59,60] are examples of vapor-phase synthesis. Figure 1 shows the different synthesis methods of ZnO nanostructures. Except for wet chemical synthesis, other above-mentioned methods have limits in either large-scale applications or substrate selection due to their obstacles such as high temperatures, expensive equipment, complexity, harvesting and toxicity [16]. In contrast, wet chemical methods favor distinct advantages such as large-scale applications, the possibility of substrate selection (organic or inorganic substrate), high yield, ease of control over morphology, uniform morphology, homogeneous size and localized growth [44,61,62,63]. Among various wet chemical methods, the hydrothermal process is regarded as one of the most attractive methods for ZnO fabrication as it is facile to set up and is controllable, cost-effective, relatively low temperature and an environment benign process [39,48,64,65]. Many different approaches such as resistive nano heater [24,66,67], bulk heating [61,68], microcontact printing [17,63,69,70,71], inkjet printing [17,57,62,72,73] and laser-induced heating [44,46,61,68,74,75] have been developed via the hydrothermal method. These approaches require different setups, processing factors and also different ways for required factors. There are several parameters, such as laser power, seeding of the substrate, the type and concentration of precursor solution, growth duration and temperature, which influence ZnO’s morphology and shape during the growing process. Since properties of ZnO nanostructures are strongly correlated with their morphologies and shapes, it is valuable better to understand these parameters for desired applications [12,21,25]. Furthermore, even though these approaches mentioned above belong to the same family of growth, they possess different advantages and disadvantages.

Recently, comprehensive work was reported in synthesizing ZnO nanostructures for UV sensors [4,40,76]. Their majority was focusing on various synthesis methods. However, each method possesses various approaches and it was short of a detail interpretation about the mechanism of each method. As economic cost for the synthesis is under increasing challenge, a comprehensive review on each method is of great importance for the future study. To the best of authors’ knowledge, no previous review has precisely examined all of hydrothermal approaches and summarized the effect of critical parameters on the ZnO synthesis. Therefore, the current review aims on the latest approaches of ZnO hydrothermal growths and the effect of different parameters on its morphology. This paper provides an overview of the recent developments in ZnO nanostructure synthesis for UV sensors, particularly the hydrothermal synthesis. Different hydrothermal approaches are compared, and the critical parameters are discussed in detail. Lastly, the hydrothermal ZnO-based UV sensors for UV light are discussed as well. This review may provide a better comprehension of the current research status for hydrothermal ZnO-based applications.

## 2. ZnO Nanostructures’ Hydrothermal Growth

### 2.1. Different Types of ZnO Nanostructures

ZnO is a fascinating material with various morphologies. The chemical and physical characteristics vary as a function of morphology, size, shape and crystalline structures. Previous works have demonstrated that this material can be modelled on the desired shape and size [12,14,17,18,42,77].

**Figure 2:**
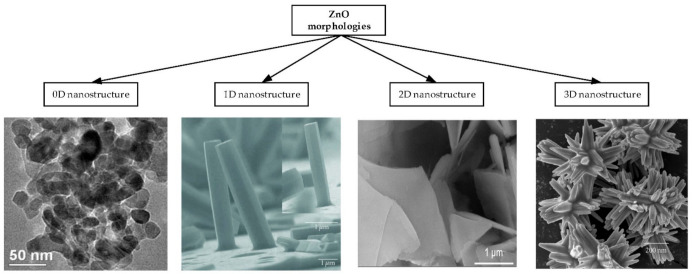
Different morphologies of ZnO nanostructures, reproduced with permission from References [46,47,64].

As shown in Figure 2, ZnO morphologies can be categorized into four classes according to their shapes:

- Zero dimensional (0D) ZnO nanostructures are those that have entered the nanoscale range in all three dimensions. They include nanoparticles [12,43,51,78] and quantum dots [19,44,79,80].
- One dimensional (1D) ZnO nanostructures are structures with at least one dimension smaller than 100 nm. They include nanowires [17,29,32,44,48,57,66,77,80,81,82], nanotubes [17,33,83], nanorods [15,22,26,72,75,77,80,84,85,86], nanoneedles [87] and nanofibers [78,88,89].
- Two dimensional (2D) ZnO nanostructures are structures in which electrons can move freely only on a non-nano scale of two dimensions, such as nanobelts [17,18,42,77,87], thin films [71,90,91] and nanosheets [46,80,88,92,93].
- Three dimensional (3D) ZnO nanostructures are built with the agglomeration of 0D, 1D and 2D nanostructures [78]. They are structures in which electrons can move freely on three non-nano scales such as nanoflowers [32,47,80,94,95], nanorings [17,18,25], nanospheres [96], nanohelix [18], nanocombs [97], superstructures [80] and sea urchin [37].

### 2.2. Hydrothermal Growth Process

**Figure 3:**
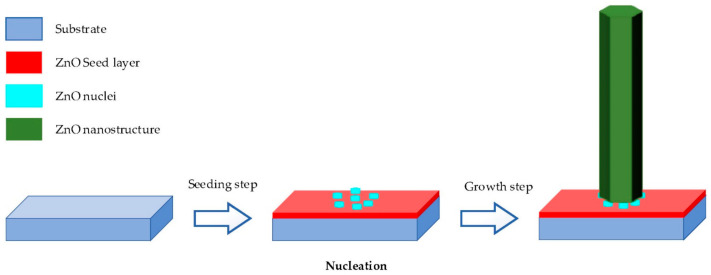
Diagram of hydrothermal process, reproduced with permission from Reference [2].

Hydrothermal method is carried out in an aqueous solution by an autoclave system [2,33,34,37,41]. It contains two typical steps as illustrated in Figure 3.

- Seeding: The subtract is seeded with a layer of ZnO nanoparticles. The seeded nanoparticles play a role in promoting nucleation for nanostructure growth by decreasing the thermodynamic barrier. The less the nucleation of ZnO is, the bigger ZnO growth and the better the crystallinity of ZnO [98].
- Growing: The seeded subtract is kept in the precursor at a certain temperature for a fixed period to ensure stable growing regimes. The precursor is a mixture of aqueous solutions containing alkaline reagent (such as NaOH, KOH and hexamethylenetetramine (HMTA)) and zinc ion salt (such as Zn (NO_3_)_2_ and ZnCl_2_) [39,40,47,99]. In addition to the precursor, a guiding agent (such as polyethyleneimine (PEI)) is inserted to decrease the lateral growth and maximize the length of nanostructures.

Deng et al. employed the hydrothermal process to grow ZnO nanorods on flexible Kapton substrate at low temperature with an equimolar solution of HMTA and zinc nitrate hexahydrate as a precursor [19]. They obtained nanorods in the form of regular hexagonal prisms with a length of 60 nm and a diameter of 100 nm. Vijayakumar et al. presented ZnO nanotubes synthesized by hydrothermal method in autoclave for 4 h at 90 °C for CO gas sensing [83]. Hu et al. hydrothermally synthesized ZnO nanowires on polyethylene terephthalate (PET) fabrics at 95 °C for 4 h [34]. The as-grown nanowires were treated hydrophobically by polydimethylsiloxane (PDMS) for lotus effect. Via hydrothermal growth, Wu et al. demonstrated the synthesis of graphene quantum dot doped ZnO superstructures for weak UV intensity sensor application at 90 °C for 2 h [80].

Typically, the hydrothermal method consists of a series of four chemical reactions. The chemistry reactions were discussed by Young et al. [40]. Firstly, HMTA was often used as a precursor and hydrolyzed to the OH^-^ and ammonia. Secondly, OH^-^ ions with Zn^2+^ ions form a complex of hydroxide of zinc Zn(OH)_2_. After then, the Zn(OH)_2_ was thermally decomposed into ZnO. These reactions are as follows [33,37,39,40,41,45,75]:

(1) $$C_{6}H_{12}N_{4}{+H}_{2}O\to {6\mathrm{HCHO}+4\mathrm{NH}}_{3}$$

(2) $${\mathrm{NH}}_{3}{+H}_{2}O\to {\mathrm{NH}}_{4}^{+}{+\mathrm{OH}}^{-}$$

(3) $${2\mathrm{OH}}^{-}{+\mathrm{Zn}}^{2+}\to {\mathrm{Zn}(\mathrm{OH})}_{2}$$

(4) $${2\mathrm{Zn}(\mathrm{OH})}_{2}\to {\mathrm{ZnO}+H}_{2}0$$

In order to control the morphology, orientation, aspect ratio and surface density of the ZnO nanostructure, parameters involved in the process must be optimized. Examples of parameters affecting nanostructure growth morphology are the pH of the solution, reagents, seed layers, temperature, guiding agents, growth time and mechanical agitations [18,50]. 

Over decades, the hydrothermal technique has proven beneficial for the synthesis of ZnO nanostructures on either rigid or flexible substrates. Rigid substrates are often concerned about glass [2,22,33,44,48,59,61], whereas flexible substrates include silicon [45,100], polyimide (PI) [44,45,48,100], PET [19,34,68], polycarbonate (PC) [48,100], Kapton [101], PDMS [17], carbon fiber [17,29] and paper [17].

## 3. ZnO Selective Hydrothermal Growth

Recent hydrothermal growths have been developed to synthesize ZnO nanostructures directly at a localized area on the substrate. These methods are called selective hydrothermal growth and were categorized into two groups: localized heat and seed patterning. Table 2 summarizes the advantages and disadvantages of these selective hydrothermal growths. 

### 3.1. Localized Heat

**Figure 4:**
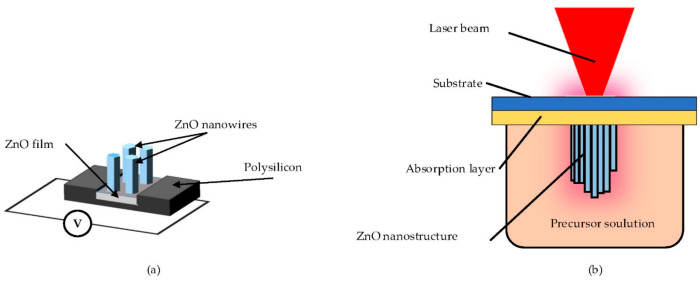
Schematic illustration of localized heat hydrothermal growth: (**a**) Joule heating, reproduced with permission from Reference [67]; (**b**) laser heating, reproduced with permission from Reference [102].

In the past decade, two main approaches of localized heat have been established for selective hydrothermal growth. These approaches include Joule heating and laser heating, as shown in Figure 4.

#### 3.1.1. Joule Heating Growth

Joule heating is based on Joule’s Law, as illustrated in Figure 4a. When the voltage is applied, current flows through the conductor. This current generates heat in the conductor owing to the presence of resistance [103]. Focused energy field (FEF) is the method based on localized joule heating at a low temperature. Besides, FEF exhibits a liquid-phase reaction at ambient pressure for selective growth. In FEF, the heat source is provided by the electric voltage. FEF can be explained as follows: (a) micro/nano heater, connected to a constant electric current, is immersed in aqueous ZnO precursors for increasing the temperature at the desired hot spot; (b) by endothermal reaction, nanomaterials are synthesized at local heated spots until the electric power is disconnected [24,66,67].

#### 3.1.2. Laser-Induced Growth

Laser heating is based on photochemical and photothermal effects [103]. When the focused laser irradiates the surface of an object, it can produce a localized temperature field [22,33,34,46,101]. Yeo et al. reported laser-induced local heating for the very first time as an alternative to joule local heating to grow ZnO nanowires for 20 min using Nd:YAG 532 nm wavelength at 130 mW laser [61]. This process is also called laser-induced hydrothermal growth (LIHG). As represented in Figure 4b, LIHG consists of a perceptible laser beam that is focused on the target subtract to increase the temperature in the system for the growth photothermally. The target substrate is immersed in a crystal-clear precursor solution. The target substrate is an underlying substrate generally covered at the top by an absorbing layer (such as gold, chromium and titanium). In this process, the temperature is firstly confined at the laser focus and then spreads radially from the absorbing layer. In their study, they prepared an aqueous solution of PEI (C_2_H_5_N), HMTA and zinc nitrate hexahydrate (Zn(NO_3_)_2_·6H_2_O) as the precursor solution. Using a similar setup, Hong et al. discovered that the optimum laser power was around 120 mW [74]. Later on, this method was proven as digital growth, even on 3D microstructures, and was successfully performed on flexible substrates [44]. Local laser heating was previously proven to be faster and more efficient than bulk heating [61,68]. Kwon et al. employed focused laser heating for localized hydrothermal synthesis of ZnO and CuO bumps, where they proved that the laser increased the reaction rate as well [68]. LIHG showed 10 times faster growth and the length of the product was 3 times larger compared to continuous bulk heating when same average of power was applied [61]. This was due to the heat confined at a small area and the growth area was also extremely small compared to the location of bulk heating. In addition, Fujiwara et al. fabricated ZnO nanorod array structures on the glass covered with 50 nm gold thin film using a 405 nm CW laser beam and a precursor solution of HMTA mixed with zinc nitrate hexahydrate [102]. Recently, Liu et al. directly integrated ZnO nanowires seeding and synthesis on silicon wafers via pulsed-laser deposition [75].

### 3.2. Seed Patterning

**Figure 5:**
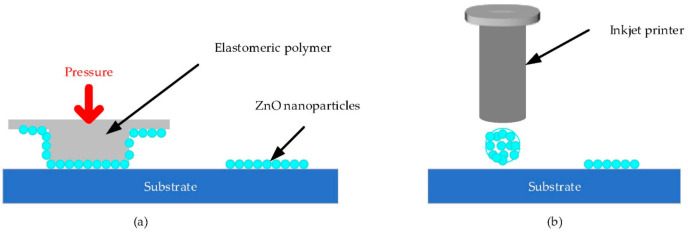
Schematic representation of seed patterning selective hydrothermal growth. (**a**) Microprinting pattern, reproduced with permission from Reference [63]. (**b**) Inkjet printing pattern, reproduced with permission from Reference [62].

Recently, various methods have been developed to deposit the seed layers for seed patterning [34,62,68]. Novel methods include microcontact printing and inkjet printing seed patterning for low temperature, as shown in Figure 5.

#### 3.2.1. Microcontact Printing

Microcontact printing is a simple patterned growth successfully developed to modify the surfaces of various textures [69,70]. Basically, this process is focused on molding and embossing with an elastomeric polymer stamp, as illustrated in Figure 5a. Firstly, the stamp was prepared for microcontact printing from the mold. This mold was obtained from oxygen plasma surface treatment and convention photolithography. Then, ZnO nanoparticle seeds were adhered to the stamp and transferred to the target substrate by pressure [17,63]. In preparation of ZnO hydrothermal nanowires, Kang et al. employed microcontact printing to deposit seed layer and obtained ZnO nanowires with lengths and diameters in the range of 2–5 µm and 130–200 nm, respectively [63]. The as-deposited seed layer was synthesized by mixing 30 mM NaOH and 10 mM zinc acetate in ethanol at 60 °C for 2 h and cooled at room temperature.

#### 3.2.2. Inkjet Printing

Figure 5b illustrates the process of inkjet-printed patterning. In this direct and digital method, a ZnO nanoparticle solution was used as the source of seeds and these nanoparticles were inkjet printed on the target substrate before immersing the sample in the aqueous solution for selective hydrothermal growth. The ink was dropped by a piezo-electric driven Drop-On-Demand inkjet head that was integrated with the CAD system. Then, it was spread arbitrarily to draw random digital patterns of ZnO nanoparticles according to the degree of heat [17]. In the work of Ko et al., a NaOH solution in 32.5 mL of ethanol (0.03 mM) was mixed slowly with a solution of zinc acetate dihydrate (0.01 mM) in 62.5 mL of methanol at 60 °C and stirred for 2 h to prepare ZnO nanoparticles with a diameter of 5–10 nm. The as-prepared nanoparticles were used to grow vertical and urchin-like nanowires at 92 °C by inkjet-printed seed patterning in solution of PEI, HMTA and zinc nitrate hydrate for 2.5 h [62]. They obtained a length and a diameter of ZnO nanowires that were approximately 10 µm and 150 nm, respectively. Ko et al. also explained that the initial diameter of the inkjet-printed ZnO seeds determined the final architecture of the ZnO nanowires. However, this inkjet printing method was shown to have a common nozzle clogging problem. Regarding this setback, researchers used zinc acetate precursor ink as the seed for local hydrothermal growth of nanowires instead of ZnO nanoparticles [57,72]. This zinc acetate seed approach not only avoids the complexity of the traditional method, but also eliminates the nozzle clogging. Sun et al. synthesized high-quality localized ZnO nanorods based on inkjet-printed zinc acetate precursor, which improved the field emission performances after eliminating the coffee ring effect [73]. The ink of Sun et al. was prepared by dissolving zinc acetate dihydrate in ethanol to obtain a concentration of 10 mM.

## 4. Influence of Fabrication Parameters on ZnO Hydrothermal Growth

**Figure 6:**
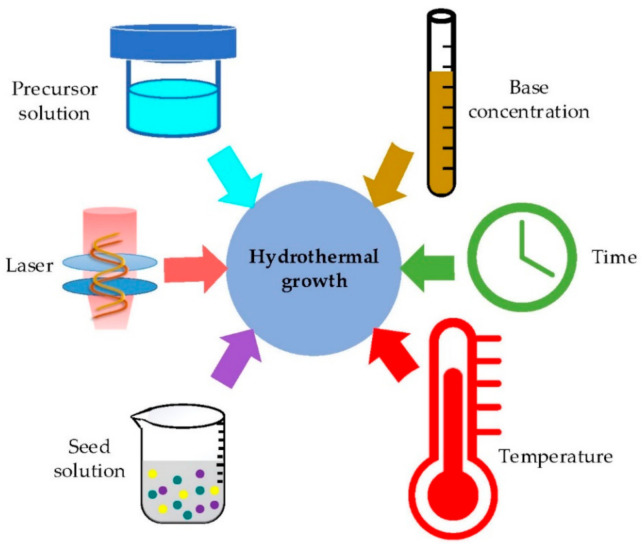
Main influencing parameters on hydrothermal growth.

The performance of ZnO nanostructures is related to their morphology and shape. Therefore, effectively controlling their shape, size and surface architecture for high yield is highly demanded. It was proven that these dimensions for nanostructure can be regulated by monitoring certain growth conditions through hydrothermal approaches [18,50,65]. As illustrated in Figure 6, these main involved parameters are laser, precursor, base concentration, growth time, temperature and seed. This section presents a comprehensive summary of the effect of these parameters on the morphology of nanostructures.

### 4.1. Influence of Laser Power

Laser beam as the key heat source has been investigated in the synthesis of nanostructures because of its high efficiency in heat transfer [22,68,74,75,95]. When the laser irradiates on the target substrate, the energy from the laser heats up electrons and then ions. At the center of the laser beam spot, the heat is at the maximum owing to the Gaussian profile of the laser beam [44,74]. However, the nanostructures synthesized can be greater than the laser spot owing to the laser beam scattering, the lateral thermal diffusion and the centrifugal extension of the nanostructures that are grown [44,61]. The spatial heat diffusion and the photothermal reaction during the growth strongly depend on the focused laser spot size. Meanwhile, it is a challenge to control the beam spot size [27,44]. Researchers have often used a process with a fixed spot size but altering the laser absorption layer. As a result, the laser-induced temperature was regulated via changing the thermal conductivity or the physical dimension (such as thickness and diameter) of the absorbing layer [44].

**Figure 7:**
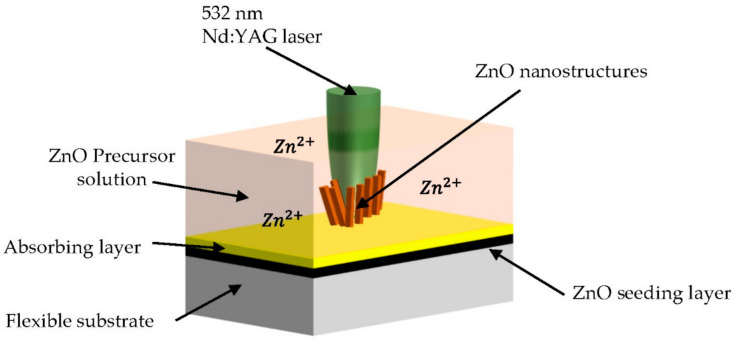
Top focusing method of laser-induced hydrothermal growth, reproduced with permission from Reference [44].

The laser plays three significant roles in hydrothermal growth. Firstly, the direction of the laser beam towards the target substrate is crucial. Two laser beam irradiation configurations are possible. The laser beam is either focused from the top on the same side of the subtract (Figure 7) or from the bottom (Figure 4b). Yeo et al. proved that higher quality of ZnO nanowires was obtained when the laser beam irradiation was on the opposite side to the growth due to a low induced temperature [61]. Fujiwara et al. also employed a similar irradiation direction to optimize the random lasing properties [102]. However, Yeo et al. successfully synthesized digital ZnO nanowires with a greater dimension than that of the laser spot directly on the growth side [44]. Liu et al. also used top focusing of the laser for the successful nucleation of ZnO nanoparticles and ZnO nanowire growth [75]. Even so, the bottom focusing case is more efficient, stable and preferable because there is no direct interaction between the nanostructures and the laser beam [61]. Secondly, it is because of the laser intensity. A higher intensity increases the growth rate because more photons are absorbed and then a higher temperature is generated. Fujiwara et al. discovered that the quality of ZnO nanorods was improved with an increase of the excitation laser intensity [102]. The higher value of the laser intensity made an increase in ZnO film quality and a decrease in sheet strength [32]. However, there was a maximum laser intensity and its excess destroyed the synthesized nanostructures [29,32,61]. Third, laser power is associated with the diameter of the laser as well. Jung et al. conducted two growth experiments to show that the laser power and beam diameter significantly influence the nanostructure morphology [104]. It was found that the laser beam with the smaller diameter produced ZnO faster and longer owing to higher laser energy at the spot center. Results showed that the growth output is proportional to laser power due to the increase of induced temperature, which plays a role of a catalyst in the chemical reactions. Furthermore, there is a minimum laser power for nanostructure growth to occur.

### 4.2. Influence of Precursor Solution

The mixture of precursor solution is mainly responsible for the chemical reaction during hydrothermal process. The careful control of initial reaction and precursor conditions gave predicted stoichiometric compositions of final arrivals. Multiple metal oxide nanostructures can be directly grown on the same substrate with consecutive hydrothermal growths with different precursor mixtures [1,2,44]. For instance, Yeo et al. applied LIHG twice with separate precursors to synthesize ZnO and TiO_2_ nanowires on the same PI substrate [44]. As mentioned in Table 3, precursors play an important role on the size of the nanostructures.

The change of the precursors significantly influences the output results [65,78,98]. Naif et al. investigated the effect by altering zinc acetate dehydrate, zinc nitrate hexahydrate and zinc chloride [65]. Their finding was that the zinc salt precursors significantly influence the aspect ratio and the morphology of nanowires.

Adequate control over the chemical reactants was utilized to regulate morphologies and the size of the final ZnO-grown nanostructures [31,32,64,78,106]. By varying the concentration of the reactants from 0 to 400 mM, Amin et al. demonstrated that the length, diameter and density of the ZnO nano/micro rods were also varied [64]. According to their findings, nano/microrod, wire-like rods and ultrathin nanowires can be obtained by adjusting the precursor concentration. There is limit for only the radial growth when the concentration is continuously increased. The rods became thin film at higher concentration. Additionally, Yeasmin et al. proved that nanorods synthesized under low precursor molarity resulted in thinner rods [31].

**Figure 8:**
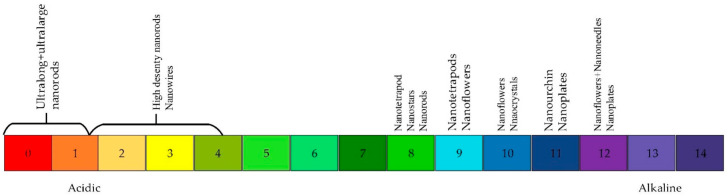
ZnO morphology versus pH of precursor.

The pH value of the precursor mixture has an important influence on the output products for the growth of nanomaterials with chemical route [39,64,65,89,104]. When the pH increased by adding ammonium, ammonia hydrolyzed into NH^4+^ through Equation (2) and Zn(OH)_2_ rose according to the increase of OH^-^ concentration in the solution described by Equation (3). When the pH was decreased by adding HCl or HNO_3_ (pH < 7), the dissolution of the precipitates occurred through Equation (3) followed by the rise of formation of Zn^2+^ ions leading to larger and longer nanorods. According to Amin et al.’s work, morphologies and sizes were varied with the pH and there was a critical pH value where there was etch instead of growth [64]. Figure 8 shows the variation of ZnO morphologies with different pH of the precursor solution. ZnO nanorods were obtained at pH = 6.6, nanotetrapod ZnO was obtained at pH = 8, flower-like structures were obtained at pH = 9.1, urchin-like structures at pH = 11.2, no growth at pH < 1.8 and ultralong and ultra-large nanorods at a pH between 1.8 and 4.6 [64]. Young et al. also synthesized nanowires, nanostars and nanoflowers at pH = 6.53, 8.18 and 9.18, respectively [107].

### 4.3. Influence of Base Concentration

To get desired nanostructure morphologies, base solutions such as NH_4_, NaOH and KOH were added to the growth solution [17,18,20,41,99]. It was proved that a supersaturated solution in Na_2_CO_3_ was a significant factor for the synthesis of nanowires due to its action as a weak base hydrolyser in the water solution and producer of OH [77]. Naif et al. experimented with the base concentration of 2.044 M, 2.673 M, 3.145 M, 4.193 M and 4.717 M to scale up the fabrication of nanowires [65]. According to their findings, the synthesis of nanowires was started when the base was above the saturation concentration of Na_2_CO_3_ (in their case, the saturation level was 2.673 M). They also found that an increase of Na_2_CO_3_ concentration caused the enhancement in the length of ZnO nanowires, but a decrease in diameter because higher Na_2_CO_3_ concentration resulted in the side facets becoming positively charged and suppressing lateral growth. Altering the concentration of base solutions in the aqueous solution resulted in ZnO nanostructures with different properties [16,89]. Lu et al. hydrothermally achieved the growth of ultralong nanobelts with honey-like micropattern on the Zn foil at 150 °C with concentrations of NaOH and (NH_4_)_2_S_2_O_8_ of 2 M and 0.4 M, respectively [108].

With cautious adding ammonium hydroxide NH_4_OH in the precursor mixture, Boubenia et al. discovered a possibility of enhancing the nucleation sites, which led to the control of nanowires’ electrical properties and expanded the applications for flexible and electromechanical devices [16]. Based on their work, they explained the density-controlled synthesis growth mechanism of ZnO nanowires as follows: The amount of NH_4_OH had a straight effect over the concentration of Zn (II) complexes, which would significantly impact the Zn solubility in the solution. Thus, the supersaturation of precursor solution was controlled as well as the quantity of nuclei over the target substrate.

### 4.4. Influence of Growth Time

In hydrothermal growth process, growth time is another important critical parameter of ZnO nanostructures [48,65,87,97,99]. As shown in Table 3, researchers fabricated ZnO nanostructures with different growth times to set up the relationship between yield of growth and their morphologies. 

**Figure 9:**
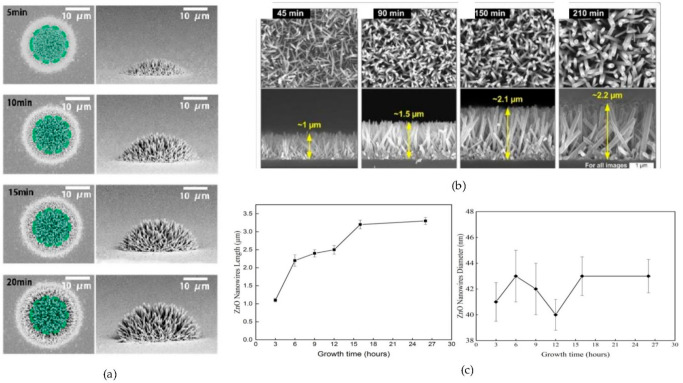
ZnO nanowires synthesized by hydrothermal method at different growth time. (**a**) SEM images of diameter, reproduced with permission from Reference [44]; (**b**) SEM images of length, reproduced with permission from Reference [100]; and (**c**) length and diameter versus growth time, reproduced with permission from Reference [65].

In laser-induced growth, nanorods’ diameter became larger and their density became less with the increase of growth time, but the nanorod lengths were almost unchanged with the growth time [22,102]. Control over the growth time enabled only an optimization of the radial growth of ZnO nanowires, as indicated in Figure 9a [44,61,74]. This phenomenon is due to the nucleation and growth theory, where rather than forming new nanostructures, ZnO was successively deposited on the preformed ZnO nanostructures. Initially, it was the absorbing layer that heated the precursor solution. At a certain level of the precursor solution’s temperature, the continuous formed ZnO components were oversaturated and resulted in a trigger of the nucleation process. Then, the ZnO nanostructures were nucleated and the growth on the hot area of the absorbing layer was taken place. Once the nucleation began, the growth of ZnO nanostructures consumed the ZnO component in the precursor solution and this avoided further oversaturation of ZnO components. Thus, it became impossible to form new ZnO nanostructures nuclei to enhance the packaging density [22].

The growth time monitors the aspect ratio of ZnO morphology. In hydrothermal growth, the length of nanorods was continuously and steadily increased with the increase of time, as shown in Figure 9b [64,65,100]. Above a specific time, there was no further expansion of the length due to a closure–precipitation equilibrium. Amin et al. attributed this observation to the fact that at a certain time, the OH^-^ was consumed due to the hydrolyze of OH^-^ in water from HMTA, leading to the termination of the reaction [64]. Naif et al. successfully synthesized ZnO at different growth times from 3 to 26 h in the following conditions: 140 °C with 4.193 M Na_2_CO_3_ and 0.032 M ZnCl_2_. As shown in Figure 9c, the results firstly emphasized that the length of ZnO nanowires increased from 1.1 to 3.3 µm with a prolonged growth time due to an Ostwald ripening process, while the diameter was kept constant around 41 nm. Secondly, the length of ZnO was almost constant from 16 h since Zn^2+^ ions were consumed in the mixture [65]. By further increasing the time, ZnO nanorods continually grew longer along the (001) direction and became wider along the (010) direction, whereas, with higher growth durations, the diameter decreased [106,108]. These results pointed out that the ZnO nanostructures grew only along the c-axis after the initial nucleation. The mechanism responsible for this exclusive axial growth may be the alkaline condition of the precursor solution. This mechanism was due to track ions such as acetate ions and/or carbonate ions. In fact, when the precursor solution was alkaline, the (1120) side facets and the (0001) end facets of ZnO nanowires became positively and negatively charged, respectively. This in turn suppressed the radial growth [65].

### 4.5. Influence of Growth Temperature

The hydrothermal method occurs at low temperatures and in an autoclave environment. Therefore, the temperature also has a significant role in controlling the structure morphology. Table 3 mentioned the effect of temperature on the geometry of ZnO. 

The role of the temperature is to heat the mixture of precursors in autoclave to activate the chemical reactions. For example, the growth reaction was accelerated at higher temperatures because an increase of OH^–^ ion concentration was caused by the faster decomposition of HMTA at higher temperatures [104]. The range of appropriate temperature growth depended on the different semiconductor materials. For instance, the range of proper temperature for TiO_2_ nanowires (120–180 °C) was higher than for that of ZnO nanowires (60–120 °C) [44]. 

The temperature was found to control the size of ZnO nanostructures [64,65,73,78]. To investigate the influence of the temperature on ZnO nanowires, Amin et al. conducted a hydrothermal growth of nanorods in aqueous solution with a constant pH (pH = 6.6), specific growth time (t = 5 h) and a fixed precursor concentration (100 mM), and the growth temperature was changed from 50 °C to 110 °C [64]. Their results indicated that the ZnO nanorods’ aspect ratio increased with the temperature up to 95 °C but no further enhancement was found in the aspect ratio above 110 °C. Through their findings, they affirmed that the feasible temperature for growth was less than 100 °C. There was an optimum temperature where a high density of ZnO nanowires grew in a uniform and conform fashion [100]. Naif et al. carried out similar work at higher temperatures (at 120 °C, 140 °C and 160 °C) and discovered that the high yield of growth was centered around 140 °C (diameter and length are 50 nm and 1 µm, respectively) [65].

The type of synthesized ZnO nanostructures is also affected by the temperature. On the Zn foil substrate, Lu et al. synthesized well-aligned ZnO nanorods of 30 nm in diameter and 200 nm in length at 22 °C and ultralong ZnO nanowires arrays with honeycomb-like structures of 60 to 200 nm in diameter and 10 to 30 mm in length at elevated temperature under similar conditions [108].

In hydrothermal method, ZnO nanostructures were treated with thermal annealing after either the seed deposition or the growth in order to alter their properties [1,106,109,110]. For instance, Filip et al. reported a significant difference in crystalline structure on a seed layer between annealed and non-annealed substrates [35]. Lupan et al. demonstrated that post-treatment thermal annealing led to improvement in the crystallinity and the performance of ZnO nanomaterials [82]. Sandeep Sanjeev and Dhananjaya Kekuda also showed that annealing temperature affected the structural and optical properties of the ZnO thin film [36]. Wahid et al. reported that the optimum annealing temperature was 150 °C, where they obtained high-resistant ZnO nanorods with a length and diameter of 4000 nm and 379 nm, respectively [86]. They also discovered that the ZnO growth rate depended on the annealing temperature, as vertical nanorods were observed below 150 °C and ZnO homocentric bundles on the vertical nanorods above 150 °C. Through careful analysis of the seed layer, Wahid et al. explained the mechanism behind this observation as follows. At annealing temperatures above 150 °C, more energy was present in the seed layer, which enhanced the kinetic energy of the seed layer molecule. In consequence, the molecular motion increased and this caused the seed layer to stretch more and reduce the surface tension. As a result, the seed nanoparticles agglomerated, which then brought the nanoparticles together during the annealing process. As the seed nanoparticles were agglomerating, the active nucleation sites of ZnO seed were disorientated, resulting in multifarious ZnO nanorod growth orientation, which promoted bundling of the ZnO nanorods [86,111]. Meanwhile, Wei et al. reported that this agglomeration phenomenon happened because annealing produced the dried organic compound (diethanolamine) [112].

### 4.6. Influence of the Seed Solution

The seed layer condition and coating are critical for high-yield ZnO nanostructures’ growth from the initial crystal nucleation [17,100]. ZnO nanowires and nanorods were unsuccessfully grown without seed coating [95,100]. Asib et al. discovered that bigger and less distributed ZnO nanorods were synthesized on glass without a seed layer compared to a compact and denser distribution of thin ZnO nanorods synthesized on glass with a TiO_2_ layer because the glass did not provide the nucleation for nanostructures to grow [2]. Yoo et al. hydrothermally synthesized a conform and uniform large area of ZnO nanowires using ZnO nanoparticle seeds and Ag seeds [100]. They explained that the Ag-seeded substrate needed a longer time and higher temperature (130 °C) compared to the ZnO-seeded substrate for the same length.

In the work of Farhad et al., they revealed that pre-depositing ZnO seed layer was beneficial for synthesizing aligned ZnO nanorods along the c-axis direction parallel to the substrate due to the high relaxation [95]. They also showed that the optical characteristics, such as reflection and transmission of ZnO nanorods, were enhanced. Still, the optical bandgap stayed unchanged when nanorods were prepared on seeded soda line glass (SLG) and fluorinated tin oxide (FTO) compared to those prepared on a non-seeded layer. The reason was that the pre-deposition of the ZnO seed layer promoted thicker and denser ZnO nanostructures. In the same perspective, Filip et al. also demonstrated the similar impact of seed treatment on the structures, morphology and optical properties using a one-step hydrothermal process [35]. Their results showed a successful synthesis of ZnO nanorods and nanoplates with homogeneous configuration on seeded glasses with three different seeded layers (zinc acetate, ethanolamine and 2-methoxyethanol). Filip et al.’s nanostructures provided excellent antireflection properties with an enhancing of transmittance up to 90% and a reduction of the bandgap energy to 3.22 eV.

Lower concentrations of seed solution result in better crystallinity of ZnO. Eom et al. reported that the shape of the ZnO structures changed to nanowires while the crystallinity was poorer and the size of the crystalline was smaller when the seed solution to growth solution (S/G) ratio was increased. Nanorods with the best crystallinity and wurtzite structure were successfully achieved with S/G = 1:10 [98].

## 5. ZnO-Based UV Sensors

**Figure 10:**
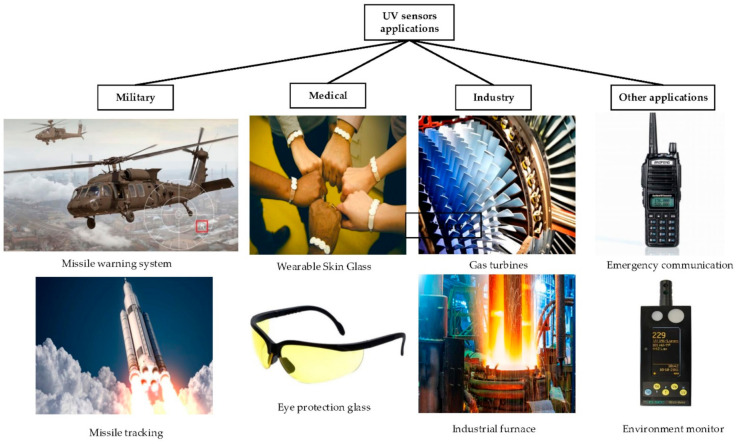
Application of UV sensors (military, medical, industrial and other applications).

UV sensors have great applications, including flame detection, UV communication, UV calibration and monitoring, missile tracking, astronomical science, industrial production and healthcare, as classified in Figure 10 [3,4,17,19]. Therefore, a strong and high demand for the excellent performance, high stability and a simpler fabrication process in UV detection systems is increasing. This requires excellent photoresponsivity in the UV wavelength bands [19]. Based on the working principle of UV sensors, ZnO-based UV detectors can be summarized in five types, including photoconductive, metal-semiconductor-metal (MSM), Schottky barrier, p-n and p-i-n junctions [4,5]. Zou et al. detailed the explanation of the mechanism, and the advantages and the disadvantages of each type [4]. 

The properties of ZnO, including piezo-phototronics and piezotronics, are of great interest for the realization of UV sensors [38,60,113,114]. The piezo-phototronic effect of ZnO boosted ZnO-based UV sensors through coupling photons with semiconducting, optical and piezoelectric properties in the material [81,115]. Particularly, the strain/stress-induced piezoelectric polarization charges may tune one or more of the procedures during the photon–semiconductor interaction, such as photon-excited carrier creation, separation, transport and recombination at the p–n junction and metal-semiconductor (M–S) contact [10,93]. Besides, the piezo-phototronic effect may provide tunable optoelectronics by using piezoelectric polarization charges as the gate bias to regulate the interface of the Schottky barrier or electronic energy states. On the other hand, piezotronics couples the semiconducting and piezoelectric properties of ZnO through stress/strain [11,38,113]. The polarity and magnitude of the piezoelectric potential within ZnO depend on the polar direction of the crystal as well as the magnitude and direction of the mechanical actuation. The generated piezoelectric potential can tune the Schottky barrier at the M–S interface by altering the redistribution of free carriers during the contact [93]. Recently, Boruah clarified the plasmonic, piezo-phototropic and phototropic effects of ZnO for the optimization of the performance of UV photodetectors (PDs) [76]. He also classified UV PDs in two categories: conventional and self-powered UV PDs. Conventional UV PDs include MSM and the Schottky junction. Self-powered UV PDs include the Schottky junction, p-n junction and photoelectrochemical cells.

In this work, all the above-mentioned sensors are classified on the basis of the composition of ZnO nanostructures. This classification consists of two groups, including pure ZnO nanostructures and ZnO composites. In this section, we elaborate on full insight into ZnO UV applications.

### 5.1. Pure ZnO Nanostructures for UV Sensing

**Figure 11:**
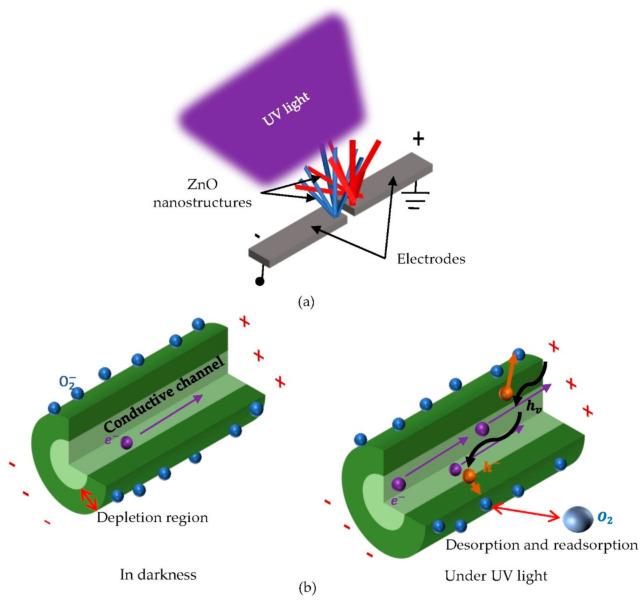
ZnO nanostructure-based UV sensor. (**a**) Schematic diagram of UV sensor with ZnO nanostructures, bridge network and (**b**) sensing mechanism, reproduced with permission from Reference [75].

It is known that the conductivity of ZnO nanostructures changes when they are exposed to UV light [61,72,74,88]. Numerous works reported that two ZnO nanostructures arrays connected as a bridge network could be used for MSM UV detection during the last decade, as shown in Figure 11a [20,24,72]. The longer the nanostructures were, the better they were connected [61]. Consequently, the sensitivity of the device increased. For instance, Yeo et al. demonstrated the applicability of ZnO nanowires on 3D structures for use in MSM PDs [61]. The current of Yeo et al.’s sensor with the UV light was approximately 11 nA under 1 V external bias. Kwon et al. designed a similar UV sensor using ZnO hemispherical bumps, but with the photocurrent of 100 nA at a 1 V bias voltage [68]. Table 4 summarizes the UV sensing property of pure ZnO with different morphologies.

The principle of the MSM UV sensor is illustrated in Figure 11b. In the dark environment, the oxygen molecules capture the free electrons to form a depletion region. Under UV illumination, ZnO absorbs UV light to excite electrons in the valence band to the conduction band and generate electron–hole pairs. Amongst these photo-induced hole pairs, holes are trapped at the oxygen-related hole-trapping states. Generally, these states exist at the surface of the nanostructure. Since the number of electrons and holes is unbalanced, the recombination rate decreases. The remained electrons are either re-adsorbed to the oxygen molecules at the surface, aggregated at the anode or recombined with the ionized hole. The sensor mechanism is described through the three reactions as follows [23,61,68,74]:

(5) $$O_{2(g)}{+e}^{-}\to O_{2(\mathrm{ad})}^{-}$$

(6) $$\mathrm{hv}\to h^{+}{+e}^{-}$$

(7) $$h^{+}{+O}_{2(\mathrm{ad})}^{-}\to O_{2(g)}$$

For the light with shorter wavelength than the bandgap of ZnO, the photo-generated holes combine with adsorbed oxygen ions and thereby are detached from the surface of ZnO nanostructures. Furthermore, the conductivity is also increased by these photo-generated electrons due to the increase in density of electrical carriers [68,74].

By controlling the laser power and growth time, Hong et al. easily integrated ZnO nanowires into the prefabricated electrode layers to form a photoconductive channel [74]. They used LIHG twice for bridging the gaps between the two electrode pads to demonstrate a photoconductive UV sensor. Their sensor displayed a significant increase of current by 2 to 3 orders of magnitude when exposed to UV light at a fixed voltage, which showed an apparent response to UV light. Comparing the performance of sensors with single, double and triple ZnO nanowires, Hong et al. discovered that the triple junction showed the best photoresponsivity with rising times of 4.2 s and 29.32 s and decay times are of 4.41 s and 38.6 s, respectively.

Powerful UV devices have also been designed on flexible material through hydrothermal methods because of their low temperature. Yang et al. employed FEF-synthesized nanowires to design ZnO-based devices with outstanding UV and gas sensing as well as excellent robustness and mechanical loading conditions on a PI substrate [24]. At a 1 V bias, the current of the designed sensor was significantly enhanced from 240 pA in the dark to 24.1 nA under UV light. Their sensor showed fast responsivity, high sensitivity and good repeatability. Furthermore, Samoucco et al. fabricated sensors that successfully detected UV light with a responsivity of 2 and 92 nA/W at a bias of 1 V for PEI and PI substrates, respectively [32]. They selected and deposited flower-like and rod-like ZnO nanostructures with a higher surface-to-volume ratio through drop-casting on the electrodes, which, in turn, were optimized to improve the electrical properties via varying the laser power and speed. Additionally, their PI sensor proved to operate under strain and be stable after long hours of operation.

Recently, the excellent control over the concentration of solutions (either seed solution, precursor solution or both) has significantly resulted in fabrications of higher-quality UV devices. Nagpal et al. manufactured a cost-effective and non-degradable UV sensor, corresponding to the WHO standard UV sensor [106]. They hydrothermally synthesized ZnO nanorods on ITO electrodes with varying precursor concentrations and growth durations for the device. They reported that the sensitivity and the response speed of UV device decreased with increases in both precursor concentration and growth time. Nanorods with the best morphology were obtained with from 5 mM concentration and 3 h growth duration (vertical nanorods and an aspect ratio of 6). Their UV sensor showed a good response under sunlight with a sensitivity of 118, rise time of 35 s and decay time of 65 s. Eom et al. studied four different values of S/G (0.1, 4, 10 and 20) to investigate their effect on the electrical properties and reactivity of UV light sensors [98]. This study discovered that increasing the molecular concentration of the growth solution allowed them to grow ZnO in the shape of nanowires rather than nanorods. Moreover, the photocurrent, response and UV sensitivity of the sensor worsened because the increase of seeds increased the nucleation site, which limited the growth of ZnO and further degraded the crystallinity of ZnO. In addition, their study showed that good crystallinity of ZnO nanorods controlled the high maximum photocurrent and response of UV sensors because they acted well as an electron transport layer. A UV sensor with a higher photocurrent, higher reactivity and shorter response time was fabricated with the ratio S/G equal to 1:10.

To design sensors with pure ZnO nanostructures, Qazi et al. prepared ZnO nanorods at a low temperature (90 °C) on pre-molded seeded substrates by a selective hydrothermal method [23]. The as-prepared nanorods formed a bridge between two microspace electrodes without destroying the electrode interface and geometry. Sensors showed fascinating performances such as low power, fast sensitivity and reproducibility. The designed sensor was in high gain (3.11) and its sensitivity was 2 A/W at 5 V applied voltage. The response and recovery times were 72 s and 110 s, respectively.

In order to prove the improvement of UV sensors via heterojunction, scientists fabricated pure ZnO-based UV sensors with an acceptable speed response to UV light [2,9,26,110]. For instance, Liu et al. designed a pure ZnO nanowire-based UV PD on Si substrate with sensitivity lower than that of a graphene/ZnO one (1.46 compared to 10.71) by growing nanowires at 120 °C [9]. Noothongkaew et al. also prepared a pure ZnO nanorod that showed a lower UV response (2 × 10^4^ A/W) to 365 nm UV light compared to CuO/ZnO composite one (8 × 10^4^ A/W) through their investigation [26].

### 5.2. ZnO-Based Composites for UV Sensing

Hybrid ZnO with other materials enhanced the sensing property of ZnO-based sensors [12,17,21,33,41,71]. This enhancement is due to two reasons. Firstly, combined materials form the heterojunction, homojunction and Schottky junction, which reduce the grain boundaries’ barrier and allow electrons to migrate easily inside the material. Secondly, there are synergistic effects among the different components of the material composite [4,12,21,116]. These combined materials include hetero-elements (such as Ni, Cu, Pt…etc.), metal oxide (such as CuO, SnO_2_ and In_2_O_3_), carbon materials (such as carbon nanofiber and carbon nanotube) and two-dimensional materials (such as GaN, SiC and graphene) [4,7,25,28,79,99,116]. Table 5 summarizes the UV sensing performance of ZnO composites materials. Here, we discuss ZnO-based composite UV applications. 

To fabricate a transparent and flexible UV sensor, Yoo et al. successfully developed low-temperature Ag-seeded hydrothermal nanowires grown on flexible plastics such as PET and PC [100]. Under UV light, the role of Ag electrodes was to collect the free electrons separated from the generated excitons inside the ZnO nanowires, resulting in a photocurrent signal [85]. The investigation of these UV sensors showed a clear response to UV power density. They also optimized the sensing performance as well as device transmittance by adjusting the ZnO nanowires’ Ag structures via the variation of growth time and temperature [100].

Using a composite detection structure consisting of ZnO nanorods and nanofiber, Feng et al. reported a simple, efficient and cost-effective UV light detection configuration with wavelength selectivity [89]. Via their experimental work, they discovered OH^−^ as the key factor in the hydrothermal growth of ZnO nanorods. When the concentration of OH^−^ was varied between 0.01 and 0.03 M, ZnO nanorod films with different configurations were obtained. The OH^−^ concentration of 0.015 M was reported the most appropriate condition of ZnO nanorod growth and nanorods with higher density, uniformity and higher specific area were obtained. 

For the hydrothermal growth of ZnO pyroelectric nanowires, Dong et al. prepared a mixture of 0.02 mM HMTA and 0.02 mM zinc nitrate [60]. Before nanowire growth, they deposited a 100 nm thick layer of ZnO seed on the p-Si substrate by the radio-frequency magnetron sputtering technique [19,21,59]. The as-grown nanowires were uniform with diameters of 40–70 nm and lengths of approximatively 2 µm. The nanowires of Deng et al. were used to construct self-powered p-Si/n-ZnO heterojuncted UV PD [60]. Without externally applied voltage, the PD had a uniform and stable UV detection capability with outstanding photoresponsivity. Furthermore, the as-prepared UV PD showed the relationship between the pyro-phototronic effect and the temperature. At a lower ambient temperature, the temperature variation (T) caused by UV light varied more than that of at room temperature (RT), which reduced the responsivity of the UV PD via the pyroelectric effect. At 77 K, the photoresponse of the UV PD improved by more than 1304%, whereas at RT, the response only improved by 532.6%. The UV PD worked perfectly even if the temperature was raised to 85 °C. When the temperature was 85 °C, the photoresponse of the sensors significantly increased by more than 567% because of the pyro-phototronic effect.

Metal oxides also proved to be efficient hybrid materials to improve the quality of ZnO-UV devices [116]. Asib et al. presented Ti0_2_/ZnO nanorod thin films for manufacturing faster response and high-sensitivity UV sensors via the hydrothermal technique [2]. During their manufacturing, they prepared different samples of TiO_2_ seed layer, ZnO and TiO_2_/ZnO nanorods as the base material for UV sensors. Investigations showed the sensitivities of the fabricated sensors were 9.2, 1.5 and 1.0 for TiO_2_/ZnO nanorods, TiO_2_ seed layer, and ZnO rods, respectively. Furthermore, the responsivities of these sensors were 1.70 × 10^−1^ A/W, 7.76 × 10^−5^ A/W and 2.22 × 10^−7^ A/W for TiO_2_/ZnO nanorods, TiO_2_ seed layer and ZnO rod-based UV sensors, respectively. In their work, the smaller and denser nanorods gave better performance of the UV sensor due to the higher surface sensing area of the fabricated ZnO nanorods. The results also indicated that TiO_2_/ZnO nanorods provided the most outstanding response, sensibility and stability to UV light owing to a capacitive and absorbance-assisted layer on the glass surface formed by tiny nanorods and seed layers. Similar work was done by Noothongkaew et al. to design CuO/ZnO nanocomposite-based UV PDs with high responsivity and good reliability compared to pure ZnO nanorod ones [26]. AlZoubi also reported an enhancement of UV detection of a ZnO nanowire/graphene oxide composite-based sensor compared to pure ZnO nanowire-based sensor [8]. The sensitivity of AlZoubi’s sensor was about 1.7 at a bias voltage of 5 V.

To prove the significant influence of the seed layers on the UV light sensing performance of ZnO-based UV heterojunction devices, Ozel et Yildiz successfully prepared SnO_2_ nanocones and nanorods on ZnO and TiO_2_ seed layers and employed UV photodiodes SnO_2_/ZnO/p-Si and SnO_2_/TiO_2_/p-Si for heterojunction [1]. Despite the fact that both devices showed relatively high performance, the SnO_2_/TiO_2_/p-Si UV photodiode exhibited the highest UV sensing performance. The authors reported that the outstanding performance of SnO_2_/TiO_2_/p-Si UV photodiode was owing to the greater density of synthesized nanostructures and the increased UV irradiation harvesting.

Doping and loading of donor elements into ZnO structures have been widely studied to improve the stability, photoelectrical and optical properties of ZnO nanostructures [12,17,19,28,40,59,99]. Tsay et al. showed that the co-doping of Ga and In ions into ZnO crystal significantly improved the photoelectrical properties and increased the p-n heterojunction UV PDs’ stability more than single doping of Ga or In [110]. They further explained that the simultaneous doping of In and Ga enhanced the carrier concentration, increased the charge transport ability and compensated for the dopant-induced lattice distortions. Shen et al. also demonstrated the improvement of the UV sensitivity of their ZnO nanofiber UV sensor after the doping of Ag via a hydrothermal process [88]. 

ZnO nanomaterials combined with 2D materials are also an interesting way to improve UV devices [9,17]. Lee et al. fabricated UV sensors based on dimensional-hybrid nanostructures such as nanowires, nanostars and nanoflowers. Excellent photoresponse and mechanical properties were achieved in these sensors [107]. In their work, they employed an efficient hydrothermal growth for the synthesis of hybrid ZnO nanostructures/2D materials (graphene and MoS_2_). With increasing pH (nanowires < nanostars < nanoflowers), the photocurrent of the device was enhanced regardless of the 2D materials, which can be understood by the increase in the density of the oxygen vacancies in ZnO nanostructures induced by pH-mediated structural tailoring. Shen et al. prepared ZnO composite graphene for the fabrication of high-sensitivity UV sensing [7]. The results showed that the sensitivity of the ZnO composite graphene-based UV sensor (427.76 pm/nW·cm^2^) was enhanced by nearly 20% compared to ZnO nanosheet-based sensor (357.85 pm/nW·cm^2^). Goswami et al. also deposited graphene quantum dots on ZnO nanowire/GaN nanotower composites for the manufacture of a very powerful heterojunction UV PD [79]. The device of Goswami et al. exhibited outstanding responsivity, excellent switching speed and lower signal detection.

## 6. Conclusions and Perspectives

With continuous exploiting new and high-yield hydrothermal processes for ZnO synthesis, novel and diverse morphologies have been found as well, which has further broadened their UV sensing applications. Hydrothermal growth is regarded as one of the more efficient and easy methods for large-scale applications. Via hydrothermal growth, various ZnO morphologies are synthesized, including 0D, 1D, 2D and 3D morphologies. Special attention has been given to achieve the desired morphology via various parameters. Variants such as laser, growth duration, temperature, precursor, pH and seeding solution were summarized based on their effects on the morphology. Increasing the growth duration has been found to increase the axial and radial growth of the nanostructures. The laser, seed solution, precursor and temperature of growth were shown to change the architecture of the ZnO nanostructures from 1D to 2D and even to 3D structures. Higher pH (pH > 8) has been proven to favor the growth of different geometries such as nanorods, nanocrystals, nanoplates, nanoflowers, nanostars, nanotetrapod-like and urchin-like structures.

As piezo-phototronic and piezotronic materials, ZnO hydrothermal nanostructured have been explored for the design of conventional and self-powered UV sensors with outstanding sensitivities and photoresponse speeds. UV devices’ structures and working mechanisms are related to the type of UV devices. The sensitivity and the response time of these ZnO-based UV devices, which were affected by the architectures and the dimensions of nanostructures, were summarized. To enhance the efficiency of these devices, combining ZnO with other materials has been used to form heterojunction composites. These composite-based sensors give possibilities to save time as well as costs for UV applications

Even with the great success of ZnO hydrothermal nanostructures in UV technology, there are still some significant challenges.

(1) The synthesis of well-controlled ZnO nanostructures via the hydrothermal method remains uncertain. The stability of their morphology, geometry and size are varied with the experimental conditions. The precious controlling architecture of ZnO nanostructures is still challenging.
(2) Composite materials with ZnO nanostructures can regulate the defects of ZnO-grown nanostructures and enhance the quality of UV sensors. However, comparative research for these composite materials is needed.
(3) The sensitivity and the photoresponse speed of UV devices are still limited. Therefore, the improvement of their performance is of vital importance for the development of UV applications.
(4) Some preparations of ZnO-based UV devices are still inconsistent and time consuming. More affordable and easily manufactured ZnO nanostructures will be revolutionary for UV devices in the future.

## Figures and Tables

**Table 1:** Summary of ZnO nanostructure properties.

Type of Property	Property
Preparation	Easy to grow [12]. Low- and high-temperature operation capability. Architecture and property controllability. Facility of integration on either rigid or flexible devices [20].
Optical	Large bandgap [13]. Good transparency to visible light. Luminescent material [20]. Good transmissibility and reflexibility [35,36].
Electrical	Semiconductor material. Good electron mobility [37]. Good chemical stability [16]. Huge piezoelectric coefficient [11,38].
Biomedical	Excellent biocompatibility [13,20]. Excellent biodegradability [25]. Non-toxicity [33,37].
Energy	Huge excitation binding energy [16,23]. Photocatalytic material [37,39].

**Table 2:** Advantages and disadvantages of selective hydrothermal growths.

Group of Selective Growth	Type of Selective Growth	Advantages	Disadvantages
Localized heat	Joule heat-induced growth	Simple for fabrication [24]. Low temperature and single step. Less usage of energy and chemicals. Strong mechanical and electrical contact between the nanomaterials and the devices.	Difficult to apply for 3D complex system [44]. Problem of leakage current. Failure at electrical contacts.
	Laser- induced growth	Simple and rapid [44,61]. Low temperature, maskless and fully digital. Well-defined configurations. High packing density and possibility of 3D structures growth [61,66].	Limited smallest local growth spot size to several microns [66,68]. Difficulty of laser power control [44].
Seed patterning	Microcontact printing	Low temperature [63]. Simple. Freedom of substrate choice. Controllability of geometry.	Fabrication of mold [44]. Impossibility of modification of the mold once fabrication is complete [62].
	Inkjet printing	Fast, maskless and digital [62]. Low temperature and environmental benign process. Freedom of substrate. Scale up for mass production. Ease modification of the growth location due to a CAD system.	Extra step required [44,63]. Inkjet nozzle clogging problem [72]. Limited ink choice in concentration and viscosity.

**Table 3:** Advantages and disadvantages of selective hydrothermal growths.

ZnO Morphology	Starting Materials	Synthesis Temperature (°C)	Growth Time	Diameter of ZnO Nanostructures	Length of ZnO Nanostructures	References
Nanowires	25 mM of zinc nitrate hexahydrate, 25 mM HMTA and 5–7 mM PEI.	95	1 h	200–400 nm	10–12 µm	[61]
Nanowires	25 mM of zinc nitrate hexahydrate, 25 mM HMTA, 5–7 mM PEI and deionized (DI) water.	95	1 h	>20 µm	-	[44]
Nanowires	25 mM of zinc nitrate hexahydrate, 25 mM HMTA and 6 mM PEI.	-	-	-	9.9 µm	[24]
Nanowires	25 mM of zinc nitrate hexahydrate, 25 mM HMTA and 5–7 mM PEI.	90	2.5 h	100–150 nm	1–3 µm	[72]
Nanowires	25 mM of zinc nitrate hexahydrate, 25 mM HMTA and 5–7 mM PEI.	95	1 h	15 µm	200–400 nm	[105]
Hemispherical bumps	Mixture of equimolar zinc nitrate hexahydrate and HMTA.	90	5 h	400 nm	2.2 µm	[64]
Nanorods	Mixture of equimolar zinc nitrate hexahydrate and HMTA.	90	3 h	100 nm	800 nm	[106]
Nanorods	50 mL of solution containing 0.1 M zinc nitrate hexahydrate, 0.1 M HMTA and DI water.	90	2 h	70 nm	15 µm	[26]
Nanorods	Mixture of equimolar zinc nitrate hexahydrate and HMTA	90	4 h	1.2	-	[2]
Flower-like structure	Zinc acetate dehydrate and NaOH.	120	15 min	0.6 µm	5.2 µm	[32]
Nanowires	ZnCl_2_. NaCO_3_ and DI water.	140	6 h	50 nm	1 µm	[65]
Vertical aligned nanorods	Zn(CH_3_COO)_2_·2H_2_O, HMTA, absolute ethanol and distilled water.	400	-	50 nm	500 nm	[13]
Nanorods	10 mL Zn(Ac)_2_.2H_2_O in 0.1 M methanol, 20 mL NaOH in 0.5 M methanol, DI water (K_2_SnO_3_,3H_2_O, 95%), 0.75 g of urea.	150	24 h	2.8 nm	26 nm	[42]
	20 mM Zn(NO_3_)_2_ and 20 mM HMTA	90 °C for 100 min, dried for 12 h at 60 °C and annealed 1 h at 500°C.	-	290–330 nm	3.2–3.4 µm	

-: Not reported.

**Table 4:** Summary of UV sensing properties of pure ZnO nanostructures.

ZnO Morphology	UV Light (nm)	Photocurrent I_ph_(A)	Dark Current I_dark_ (A)	Responsivity (A/W)	Response/Recovery Time (s)	References
Nanowires	365	1.1 × 10^−5^	˂10^−5^	-	-	[61]
Hemispherical bumps	365	10^−4^	5 × 10^−7^	-	-	[68]
Nanowires	365	3.51 × 10^−2^	5.6 × 10^−2^	-	6.2/11	[24]
Nanowires	365	>20 I_dark_	-	-	20/40	[72]
Nanorods	300–370	-	-	2	72/110	[23]
Nanowires	-	-	-	-	4.2/4.41 and 29.32/38.86	[74]
Nanorods	Sunlight	118 I_dark_	-	-	35/46	[106]
Nanorods	365	-	-	-	50–100/35–40	[98]
Nanorods	365	2.7 × 10^−3^	2 × 10^−5^	2 × 10^4^	-	[26]
Nanorods	-	1.98 × 10^−8^	1.97 × 10^−8^	2.22 × 10^−7^	60/-	[2]
Nanoflowers	184–365	8 × 10^−4^	10^−4^	92	-	[32]
Nanowires	-	-	-	12.4 × 10^−3^	-	[110]
Nanowires	365	2.7 × 10^−6^	1.1 × 10^−6^	-	1.18/>12.1	[9]

-: Not reported.

**Table 5:** Summary of UV sensing performance of ZnO composites.

Composite Materials	ZnO Morphology	UV Light (nm)	Photocurrent I_ph_ (A)	Dark Current I_dark_ (A)	Sensitivity W/(mW cm^−2^)	Responsivity (A/W)	Response/ Recovery Time (s)	References
Single-mode fiber/ZnO	nanorods	365	-	-	7.096	-	-	[89]
Si/ZnO	nanowires	325	-	-	-	17 × 10^−3^	7 × 10^−4^/-	[60]
TiO_2_/ZnO	nanorods	-	8.92 × 10^−5^	9.31 × 10^−6^	-	1.7 × 10^−1^	50/150	[2]
CuO/ZnO	nanorods	365	11.2 × 10^−3^	2 × 10^−5^	-	8.4 × 10^4^	5/3–5	[26]
Graphene Oxide/ZnO	nanowires	365	-	-	-	10.13 × 10^3^	11.2/81	[8]
SnO_2_/ZnO	nanocones	254	-	-	-	68 × 10^−3^	-	[1]
Ga-doped ZnO	nanowires	360–400	-	-	-	23.1 × 10^−3^	10.1/17.8	[110]
In-doped ZnO			-	-	-	34.2 × 10^−3^	10.8/13.3	
Ga+In-doped ZnO			1.1 × 10^−3^	-	-	27.1 × 10^−3^	13.2/16.9	
Ag-doped ZnO	nanorods	365	-	-	4.33 × 10^−8^	-	-	[88]
Graphene/ZnO	nanoflakes	365	-	-	4.2776 × 10^−7^	-	-	[7]
Graphene/ZnO	nanowires	350	-	-	-	1.45 × 10^2^	-	[107]
	nanostars		-	-	-	3.02 × 10^2^	-	
	nanoflowers		-	-	3.5 × 10^2^	3.5 × 10^2^	-	
MoS_2_/ZnO	nanowires		-	-	-	7.91 × 10^- 6^	-	
	nanostars		-	-	8.99 × 10^−4^	1.02 × 10^−4^	-	
	nanoflowers		-	-	-	8.99 × 10^−4^	-	
GaN/ZnO Graphene	nanorods	325	4.6 × 10^−3^	9.73 × 10^−2^	-	1204	1.12/1.16	[79]
Quantum dots + GaN/ZnO			1.314 × 10^−2^	79 × 10^−2^	-	3.2 × 10^3^	159/68.7	
Graphene/ZnO	nanowires	365	3 × 10^−6^	2.8 × 10^−7^	10.71	-	1.02/0.34	[9]

-: Not reported.

## Data Availability

No new data were created or analyzed in this study. Data sharing is not applicable to this article.

## References

[1] Ozel K., Yildiz A. (2020). SnO_2_/ZnO/p-Si and SnO_2_/TiO_2_/p-Si heterojunction UV photodiodes prepared using a hydrothermal method. Sens. Actuators A Phys..

[2] Asib N.A.M., Husairi F.S., Eswar K.A., Afaah A.N., Mamat M.H., Rusop M., Khusaimi Z. (2020). Developing high-sensitivity UV sensors based on ZnO nanorods grown on TiO_2_ seed layer films using solution immersion method. Sens. Actuators A Phys..

[3] Zou W., González A., Jampaiah D., Ramanathan R., Taha M., Walia S., Sriram S., Bhaskaran M., Dominguez-Vera J.M., Bansal V. (2018). Skin color-specific and spectrally-selective naked-eye dosimetry of UVA, B and C radiations. Nat. Commun..

[4] Zou Y., Zhang Y., Hu Y., Gu H. (2018). Ultraviolet detectors based on wide bandgap semiconductor nanowire: A review. Sensors.

[5] Razeghi M., Rogalski A. (1996). Semiconductor ultraviolet detectors. J. Appl. Phys..

[6] Shi L., Nihtianov S. (2012). Comparative study of silicon-based ultraviolet photodetectors. IEEE Sens. J..

[7] Shen T., Dai X., Zhang D., Wang W., Feng Y. (2020). ZnO composite graphene coating micro-fiber interferometer for ultraviolet detection. Sensors.

[8] AlZoubi T., Qutaish H., Al-Shawwa E., Hamzawy S. (2018). Enhanced UV-light detection based on ZnO nanowires/graphene oxide hybrid using cost-effective low temperature hydrothermal process. Opt. Mater..

[9] Liu Y., Song Z., Yuan S., Xu L., Xin Y., Duan M., Yao S., Yang Y., Xia Z. (2020). Enhanced Ultra-violet Photodetection Based on a Heterojunction Consisted of ZnO Nanowires and Single-Layer Graphene on Silicon Substrate. Electron. Mater. Lett..

[10] Rai S.C., Wang K., Ding Y., Marmon J.K., Bhatt M., Zhang Y., Zhou W., Wang Z.L. (2015). Piezo-phototronic Effect Enhanced UV/Visible Photodetector Based on Fully Wide Band Gap Type-II ZnO/ZnS Core/Shell Nanowire Array. ACS Nano.

[11] Kou L.Z., Guo W.L., Li C. Piezoelectricity of ZnO and its nanostructures. Proceedings of the 2008 Symposium on Piezoelectricity, Acoustic Waves, and Device Applications.

[12] Kang Y., Yu F., Zhang L., Wang W., Chen L., Li Y. (2021). Review of ZnO-based nanomaterials in gas sensors. Solid State Ion..

[13] Ditshego N.M.J. (2019). ZnO nanowire field effect transistor for biosensing: A review. J. Nano Res..

[14] Şerban I., Enesca A. (2020). Metal Oxides-Based Semiconductors for Biosensors Applications. Front. Chem..

[15] Characterization of Zinc Oxide Nanorod. https://www.azonano.com/article.aspx?ArticleID=2509.

[16] Boubenia S., Dahiya A.S., Poulin-Vittrant G., Morini F., Nadaud K., Alquier D. (2017). A facile hydrothermal approach for the density tunable growth of ZnO nanowires and their electrical characterizations. Sci. Rep..

[17] Xu S., Wang Z.L. (2011). One-dimensional ZnO nanostructures: Solution growth and functional properties. Nano Res..

[18] Galdámez-Martinez A., Santana G., Güell F., Martínez-Alanis P.R., Dutt A. (2020). Photoluminescence of ZnO nanowires: A review. Nanomaterials.

[19] Rong P., Ren S., Yu Q. (2019). Fabrications and Applications of ZnO Nanomaterials in Flexible Functional Devices-A Review. Crit. Rev. Anal. Chem..

[20] Bagga S., Akhtar J., Mishra S. Synthesis and applications of ZnO nanowire: A review. AIP Conference Proceedings.

[21] Bhati V.S., Hojamberdiev M., Kumar M. (2020). Enhanced sensing performance of ZnO nanostructures-based gas sensors: A review. Energy Rep..

[22] Xie Y., Yang S., Mao Z., Li P., Zhao C., Cohick Z., Huang P.-H., Huang T.J. (2014). In Situ Fabrication of 3D Ag @ ZnO Nanostructures for Micro fl uidic. ACS Nano.

[23] Humayun Q., Kashif M., Hashim U., Qurashi A. (2014). Selective growth of ZnO nanorods on microgap electrodes and their applications in UV sensors. Nanoscale Res. Lett..

[24] Yang D., Kim D., Ko S.H., Pisano A.P., Li Z., Park I. (2015). Focused energy field method for the localized synthesis and direct integration of 1D nanomaterials on microelectronic devices. Adv. Mater..

[25] Beitollahi H., Tajik S., Garkani Nejad F., Safaei M. (2020). Recent advances in ZnO nanostructure-based electrochemical sensors and biosensors. J. Mater. Chem. B.

[26] Noothongkaew S., Thumthan O., An K.S. (2018). UV-Photodetectors based on CuO/ZnO nanocomposites. Mater. Lett..

[27] Paeng D., Lee D., Yeo J., Yoo J.H., Allen F.I., Kim E., So H., Park H.K., Minor A.M., Grigoropoulos C.P. (2015). Laser-induced reductive sintering of nickel oxide nanoparticles under ambient conditions. J. Phys. Chem. C.

[28] Nurfani E., Lailani A., Kesuma W.A.P., Anrokhi M.S., Kadja G.T.M., Rozana M. (2021). UV sensitivity enhancement in Fe-doped ZnO films grown by ultrafast spray pyrolysis. Opt. Mater..

[29] Deka B.K., Hazarika A., Kim J., Jeong H.E., Park Y.B., Park H.W. (2019). Fabrication of the piezoresistive sensor using the continuous laser-induced nanostructure growth for structural health monitoring. Carbon N. Y..

[30] Henley S.J., Fryar J., Jayawardena K.D.G.I., Silva S.R.P. (2010). Laser-assisted hydrothermal growth of size-controlled ZnO nanorods for sensing applications. Nanotechnology.

[31] Yeasmin M., Das T., Baruah S. (2019). Study on the hydrothermal growth of ZnO nanorods for piezotronic. ADBU J. Eng. Technol..

[32] Samouco A., Marques A.C., Pimentel A., Martins R., Fortunato E. (2018). Laser-induced electrodes towards low-cost flexible UV ZnO sensors. Flex. Print. Electron..

[33] Mai H.H., Tran D.H., Janssens E. (2019). Non-enzymatic fluorescent glucose sensor using vertically aligned ZnO nanotubes grown by a one-step, seedless hydrothermal method. Microchim. Acta.

[34] Hu J., Zhang M., He Y., Zhang M., Shen R., Zhang Y., Wang M., Wu G. (2020). Fabrication and potential applications of highly durable superhydrophobic polyethylene terephthalate fabrics produced by in-situ zinc oxide (ZnO) nanowires deposition and polydimethylsiloxane (pdms) packaging. Polymers.

[35] Filip A., Musat V., Tigau N., Polosan S., Pimentel A., Ferreira S., Gomes D., Calmeiro T., Martins R., Fortunato E. (2020). ZnO nanostructures grown on ITO coated glass substrate by hybrid microwave-assisted hydrothermal method. Optik.

[36] Sanjeev S., Kekuda D. (2015). Effect of annealing temperature on the structural and optical properties of zinc oxide (ZnO) thin films prepared by spin coating process. IOP Conference Series: Materials Science and Engineering.

[37] Kiriarachchi H.D., Abouzeid K.M., Bo L., El-Shall M.S. (2019). Growth Mechanism of Sea Urchin ZnO Nanostructures in Aqueous Solutions and Their Photocatalytic Activity for the Degradation of Organic Dyes. ACS Omega.

[38] Pan C., Zhai J., Wang Z.L. (2019). Piezotronics and Piezo-phototronics of Third Generation Semiconductor Nanowires. Chem. Rev..

[39] Ghoderao K.P., Jamble S.N., Kale R.B. (2018). Influence of pH on hydrothermally derived ZnO nanostructures. Optik.

[40] Young S.-J., Yang C.-C., Lai L.-T. (2017). Review—Growth of Al-, Ga-, and In-Doped ZnO Nanostructures via a Low-Temperature Process and Their Application to Field Emission Devices and Ultraviolet Photosensors. J. Electrochem. Soc..

[41] Baruah S., Dutta J. (2009). Hydrothermal growth of ZnO nanostructures. Sci. Technol. Adv. Mater..

[42] Jabeen M., Vasant Kumar R., Ali N. (2020). A Review on Preparation of ZnO Nanorods and Their Use in Ethanol Vapors Sensing. Gas Sens..

[43] Basnet P., Chatterjee S. (2020). Structure-directing property and growth mechanism induced by capping agents in nanostructured ZnO during hydrothermal synthesis—A systematic review. Nano Struct. Nano Objects.

[44] Yeo J., Hong S., Kim G., Lee H., Suh Y.D., Park I., Grigoropoulos C.P., Ko S.H. (2015). Laser-Induced Hydrothermal Growth of Heterogeneous Metal-Oxide Nanowire on Flexible Substrate by Laser Absorption Layer Design. ACS Nano.

[45] Syed A., Kalloudis M., Koutsos V., Mastropaolo E. (2015). Controlled hydrothermal growth of vertically-aligned zinc oxide nanowires using silicon and polyimide substrates. Microelectron. Eng..

[46] Ryu Y.Y., Kim T., Han H.S. (2019). Synthesis of porous ZnO nanosheets and carbon nanotube hybrids as efficient photocatalysts via pulsed laser ablation. Catalysts.

[47] Zhou Q., Xie B., Jin L., Chen W., Li J. (2016). Hydrothermal Synthesis and Responsive Characteristics of Hierarchical Zinc Oxide Nanoflowers to Sulfur Dioxide. J. Nanotechnol..

[48] Oh D.K., Choi H., Shin H., Kim K., Kim M., Ok J.G. (2021). Tailoring zinc oxide nanowire architectures collectively by catalytic vapor-liquid-solid growth, catalyst-free vapor-solid growth, and low-temperature hydrothermal growth. Ceram. Int..

[49] Ameer A.A., Suriani A.B., Jabur A.R., Hashim N., Zaid K. (2019). The fabrication of zinc oxide nanorods and nanowires by sol gel immersion methods. J. Phys. Conf. Ser..

[50] Demes T., Ternon C., Riassetto D., Stambouli V., Langlet M. (2016). Comprehensive study of hydrothermally grown ZnO nanowires. J. Mater. Sci..

[51] Hjiri M., Bahanan F., Aida M.S., El Mir L., Neri G. (2020). High Performance CO Gas Sensor Based on ZnO Nanoparticles. J. Inorg. Organomet. Polym. Mater..

[52] Rai P., Kwak W.K., Yu Y.T. (2013). Solvothermal synthesis of ZnO nanostructures and their morphology-dependent gas-sensing properties. ACS Appl. Mater. Interfaces.

[53] Rajagopalan P., Singh V., Palani I.A. (2016). Investigations on the influence of substrate temperature in developing enhanced response ZnO nano generators on flexible polyimide using spray pyrolysis technique. Mater. Res. Bull..

[54] Li H., Zhao L., Meng J., Pan C., Zhang Y., Zhang Y., Liu Z., Zou Y., Fan Y., Wang Z.L. (2020). Triboelectric-polarization-enhanced high sensitive ZnO UV sensor. Nano Today.

[55] Ma S., Kitai A.H. (2017). ZnO nanowire growth by chemical vapor deposition with spatially controlled density on Zn_2_GeO_4_:Mn polycrystalline substrates. Mater. Res. Express.

[56] Güell F., Martínez-Alanis P.R., Roso S., Salas-Pérez C.I., García-Sánchez M.F., Santana G., Monroy B.M. (2016). Plasma versus thermal annealing for the Au-catalyst growth of ZnO nanocones and nanowires on Al-doped ZnO buffer layers. Mater. Res. Express.

[57] Laurenti M., Verna A., Fontana M., Quaglio M., Porro S. (2014). Selective growth of ZnO nanowires on substrates patterned by photolithography and inkjet printing. Appl. Phys. A Mater. Sci. Process..

[58] Park W.I., Kim D.H., Jung S.W., Yi G.C. (2002). Metalorganic vapor-phase epitaxial growth of vertically well-aligned ZnO nanorods. Appl. Phys. Lett..

[59] Chou C.C., Shih L.H., Chang S.J. (2020). The study of humidity sensor based on Li-doped ZnO nanorods by hydrothermal method. Microsyst. Technol..

[60] Dong J., Wang Z., Wang X., Wang Z.L. (2019). Temperature dependence of the pyro-phototronic effect in self-powered p-Si/n-ZnO nanowires heterojuncted ultraviolet sensors. Nano Today.

[61] Yeo J., Hong S., Wanit M., Kang H.W., Lee D., Grigoropoulos C.P., Sung H.J., Ko S.H. (2013). Rapid, one-step, digital selective growth of ZnO nanowires on 3D structures using laser induced hydrothermal growth. Adv. Funct. Mater..

[62] Ko S.H., Lee D., Hotz N., Yeo J., Hong S., Nam K.H., Grigoropoulos C.P. (2012). Digital selective growth of ZnO nanowire arrays from inkjet-printed nanoparticle seeds on a flexible substrate. Langmuir.

[63] Kang H.W., Yeo J., Hwang J.O., Hong S., Lee P., Han S.Y., Lee J.H., Rho Y.S., Kim S.O., Ko S.H. (2011). Simple ZnO nanowires patterned growth by microcontact printing for high performance field emission device. J. Phys. Chem. C.

[64] Amin G., Asif M.H., Zainelabdin A., Zaman S., Nur O., Willander M. (2011). Influence of pH, precursor concentration, growth time, and temperature on the morphology of ZnO nanostructures grown by the hydrothermal method. J. Nanomater..

[65] Alshehri N.A., Lewis A.R., Pleydell-Pearce C., Maffeis T.G.G. (2018). Investigation of the growth parameters of hydrothermal ZnO nanowires for scale up applications. J. Saudi Chem. Soc..

[66] Yeo J., Kim G., Hong S., Lee J., Kwon J., Lee H., Park H., Manoroktul W., Lee M.T., Lee B.J. (2014). Single nanowire resistive nano-heater for highly localized thermo-chemical reactions: Localized hierarchical heterojunction nanowire growth. Small.

[67] Chen C.C., Lin Y.S., Sang C.H., Sheu J.T. (2011). Localized joule heating as a mask-free technique for the local synthesis of ZnO nanowires on silicon nanodevices. Nano Lett..

[68] Kwon K., Shim J., Lee J.O., Choi K., Yu K. (2015). Localized laser-based photohydrothermal synthesis of functionalized metal-oxides. Adv. Funct. Mater..

[69] Qin D., Xia Y., Whitesides G.M. (2010). Soft lithography for micro- and nanoscale patterning. Nat. Protoc..

[70] Von Philipsborn A.C., Lang S., Bernard A., Loeschinger J., David C., Lehnert D., Bastmeyer M., Bonhoeffer F. (2006). Microcontact printing of axon guidance molecules for generation of graded patterns. Nat. Protoc..

[71] Zhang C., Luo Q., Wu H., Li H., Lai J., Ji G., Yan L., Wang X., Zhang D., Lin J. (2017). Roll-to-roll micro-gravure printed large-area zinc oxide thin film as the electron transport layer for solution-processed polymer solar cells. Org. Electron..

[72] Kwon J., Hong S., Lee H., Yeo J., Lee S.S., Ko S.H. (2013). Direct selective growth of ZnO nanowire arrays from inkjet-printed zinc acetate precursor on a heated substrate. Nanoscale Res. Lett..

[73] Sun L., Yang K., Lin Z., Zhou X., Zhang Y.A., Guo T. (2018). Effects of coffee ring via inkjet printing seed layers on field emission properties of patterned ZnO nanorods. Ceram. Int..

[74] Hong S., Yeo J., Manorotkul W., Kim G., Kwon J., An K., Ko S.H. (2013). Low-temperature rapid fabrication of ZnO nanowire UV sensor array by laser-induced local hydrothermal growth. J. Nanomater..

[75] Liu Z., Liu S., Wu W., Liu C.R. (2019). The mechanism of controlled integration of ZnO nanowires using pulsed-laser-induced chemical deposition. Nanoscale.

[76] Deka Boruah B. (2019). Zinc oxide ultraviolet photodetectors: Rapid progress from conventional to self-powered photodetectors. Nanoscale Adv..

[77] Hu H., Huang X., Deng C., Chen X., Qian Y. (2007). Hydrothermal synthesis of ZnO nanowires and nanobelts on a large scale. Mater. Chem. Phys..

[78] Napi M.L.M., Ahmad Noorden A.F., Loong Peng Tan M., Jamaluddin H., Hamid F.A., Ahmad M.K., Hashim U., Ahmad M.R., Sultan S.M. (2020). Review—Three Dimensional Zinc Oxide Nanostructures as an Active Site Platform for Biosensor: Recent Trend in Healthcare Diagnosis. J. Electrochem. Soc..

[79] Goswami L., Aggarwal N., Verma R., Bishnoi S., Husale S., Pandey R., Gupta G. (2020). Graphene Quantum Dot-Sensitized ZnO-Nanorod/GaN-Nanotower Heterostructure-Based High-Performance UV Photodetectors. ACS Appl. Mater. Interfaces.

[80] Wu H., Ding J., Yang D., Li J., Shi Y., Zhou Y. (2020). Graphene quantum dots doped ZnO superstructure (ZnO superstructure/GQDs) for weak UV intensity photodetector application. Ceram. Int..

[81] Liu Y., Yang Q., Zhang Y., Yang Z., Wang Z.L. (2012). Nanowire piezo-phototronic photodetector: Theory and experimental design. Adv. Mater..

[82] Lupan O., Magariu N., Khaledialidusti R., Mishra A.K., Hansen S., Krüger H., Postica V., Heinrich H., Viana B., Ono L.K. (2021). Comparison of Thermal Annealing versus Hydrothermal Treatment Effects on the Detection Performances of ZnO Nanowires. ACS Appl. Mater. Interfaces.

[83] Shivaraj B.W., Manjunatha C., Abhishek B., Nagaraju G., Panda P.K. (2020). Hydrothermal synthesis of ZnO nanotubes for CO gas sensing. Sens. Int..

[84] Znajdek K., Sibiński M., Lisik Z., Apostoluk A., Zhu Y., Masenelli B., Sędzicki P. (2017). Zinc oxide nanoparticles for improvement of thin film photovoltaic structures’ efficiency through down shifting conversion. Opto-Electron. Rev..

[85] Ok J.G., Lee J.Y., Baac H.W., Tawfick S.H., Guo L.J., Hart A.J. (2014). Rapid anisotropic photoconductive response of ZnO-coated aligned carbon nanotube sheets. ACS Appl. Mater. Interfaces.

[86] Wahid K.A., Lee W.Y., Lee H.W., Teh A.S., Bien D.C.S., Azid I.A. (2013). Effect of seed annealing temperature and growth duration on hydrothermal ZnO nanorod structures and their electrical characteristics. Appl. Surf. Sci..

[87] Al-Hadeethi Y., Umar A., Ibrahim A.A., Al-Heniti S.H., Kumar R., Baskoutas S., Raffah B.M. (2017). Synthesis, characterization and acetone gas sensing applications of Ag-doped ZnO nanoneedles. Ceram. Int..

[88] Shen T., Wang J., Xia Z., Dai X., Li B., Feng Y. (2020). Ultraviolet sensing characteristics of Ag-doped ZnO micro-nano fiber. Sens. Actuators A Phys..

[89] Feng Y., Shen T., Li X., Wei X. (2020). ZnO-nanorod–fiber UV sensor based on evanescent field principle. Optik.

[90] Soltabayev B., Mentbayeva A., Acar S. (2021). Enhanced gas sensing properties of in doped ZnO thin films. Mater. Today Proc..

[91] Jeon I.S., Bae G., Jang M., Yoon Y., Jang S., Song W., Myung S., Lim J., Lee S.S., Jung H.K. (2021). Atomic-level mediation in structural interparameter tradeoff of zinc oxide nanowires-based gas sensors: ZnO nanofilm/ZnO nanowire homojunction array. Appl. Surf. Sci..

[92] Zhang K., Qin S., Tang P., Feng Y., Li D. (2020). Ultra-sensitive ethanol gas sensors based on nanosheet-assembled hierarchical ZnO-In2O3 heterostructures. J. Hazard. Mater..

[93] An C., Qi H., Wang L., Fu X., Wang A., Wang Z.L., Liu J. (2021). Piezotronic and piezo-phototronic effects of atomically-thin ZnO nanosheets. Nano Energy.

[94] Nundy S., Eom T.Y., Kang J.G., Suh J., Cho M., Park J.S., Lee H.J. (2020). Flower-shaped ZnO nanomaterials for low-temperature operations in NOX gas sensors. Ceram. Int..

[95] Farhad S., Tanvir N., Bashar M., Hossain M., Sultana M., Khatun N. (2018). Facile synthesis of oriented zinc oxide seed layer for the hydrothermal growth of zinc oxide nanorods. Bangladesh J. Sci. Ind. Res..

[96] Nundy S., Eom T.Y., Song K.Y., Park J.S., Lee H.J. (2020). Hydrothermal synthesis of mesoporous ZnO microspheres as NOX gas sensor materials—Calcination effects on microstructure and sensing performance. Ceram. Int..

[97] Gao W., Li Z. (2009). Nanostructures of zinc oxide. Int. J. Nanotechnol..

[98] Eom T.H., Han J.I. (2018). Single fiber UV detector based on hydrothermally synthesized ZnO nanorods for wearable computing devices. Appl. Surf. Sci..

[99] Türkyılmaz Ş.Ş., Güy N., Özacar M. (2017). Photocatalytic efficiencies of Ni, Mn, Fe and Ag doped ZnO nanostructures synthesized by hydrothermal method: The synergistic/antagonistic effect between ZnO and metals. J. Photochem. Photobiol. A Chem..

[100] Yoo K., Lee W., Kang K., Kim I., Kang D., Oh D.K., Kim M.C., Choi H., Kim K., Kim M. (2020). Low-temperature large-area fabrication of ZnO nanowires on flexible plastic substrates by solution-processible metal-seeded hydrothermal growth. Nano Converg..

[101] Deng W., Jin L., Zhang B., Chen Y., Mao L., Zhang H., Yang W. (2016). A flexible field-limited ordered ZnO nanorod-based self-powered tactile sensor array for electronic skin. Nanoscale.

[102] Fujiwara H., Suzuki T., Niyuki R., Sasaki K. (2016). Realization of low threshold ZnO nanorod array random lasers using a laser-induced hydrothermal synthesis. Australian Conference on Optical Fibre Technology.

[103] Xiong G., Jia J., Zhao L., Liu X., Zhang X., Liu H., Zhou W. (2021). Non-thermal radiation heating synthesis of nanomaterials. Sci. Bull..

[104] In J.B., Kwon H.J., Lee D., Ko S.H., Grigoropoulos C.P. (2014). In situ monitoring of laser-assisted hydrothermal growth of ZnO nanowires: Thermally deactivating growth kinetics. Small.

[105] Hong S., Yeo J., Manorotkul W., Kang H.W., Lee J., Han S., Rho Y., Suh Y.D., Sung H.J., Ko S.H. (2013). Digital selective growth of a ZnO nanowire array by large scale laser decomposition of zinc acetate. Nanoscale.

[106] Nagpal S., Rahul S.V., Bhatnagar P.K. (2020). Low cost UV sensor using ZnO nanorods on ITO electrodes. Eng. Res. Express.

[107] Lee Y.B., Kim S.K., Lim Y.R., Jeon I.S., Song W., Myung S., Lee S.S., Lim J., An K.S. (2017). Dimensional-Hybrid Structures of 2D Materials with ZnO Nanostructures via pH-Mediated Hydrothermal Growth for Flexible UV Photodetectors. ACS Appl. Mater. Interfaces.

[108] Lu C., Qi L., Yang J., Tang L., Zhang D., Ma J. (2006). Hydrothermal growth of large-scale micropatterned arrays of ultralong ZnO nanowires and nanobelts on zinc substrate. Chem. Commun..

[109] Uddin A.S.M.I., Yaqoob U., Phan D.T., Chung G.S. (2016). A novel flexible acetylene gas sensor based on PI/PTFE-supported Ag-loaded vertical ZnO nanorods array. Sens. Actuators B Chem..

[110] Tsay C.Y., Hsiao I.P., Chang F.Y., Hsu C.L. (2021). Improving the photoelectrical characteristics of self-powered p-GaN film/n-ZnO nanowires heterojunction ultraviolet photodetectors through gallium and indium Co-doping. Mater. Sci. Semicond. Process..

[111] Babapour A., Yang B., Bahang S., Cao W. (2011). Low-temperature sol-gel-derived nanosilver-embedded silane coating as biofilm inhibitor. Nanotechnology.

[112] Wu W., He Q., Jiang C. (2008). Magnetic iron oxide nanoparticles: Synthesis and surface functionalization strategies. Nanoscale Res. Lett..

[113] Wang Z.L., Wu W., Falconi C. (2018). Piezotronics and piezo-phototronics with third-generation semiconductors. MRS Bull..

[114] Zhang W., Jiang D., Zhao M., Duan Y., Zhou X., Yang X., Shan C., Qin J., Gao S., Liang Q. (2019). Piezo-phototronic effect for enhanced sensitivity and response range of ZnO thin film flexible UV photodetectors. J. Appl. Phys..

[115] Liu Y., Niu S., Yang Q., Klein B.D.B., Zhou Y.S., Wang Z.L. (2014). Theoretical Study of Piezo-phototronic Nano-LEDs. Adv. Mater..

[116] Rahimi K., Yazdani A. (2020). Incremental photocatalytic reduction of graphene oxide on vertical ZnO nanorods for ultraviolet sensing. Mater. Lett..

